# Restoration of Primary Anterior Teeth with Glass Fiber-Reinforced Post and Core: 3-Year Follow-Up Case Report

**DOI:** 10.1155/2021/5537437

**Published:** 2021-05-10

**Authors:** Salah Afraa, Hashim Raghad, Khalid Ayam, Hamid Aeshah

**Affiliations:** ^1^Growth and Development Department, Ajman University, UAE; ^2^Basic Science Department, Ajman University, UAE

## Abstract

**Background:**

Esthetic appearance of primary anterior teeth is one of the major demands in the dental field. Destructed anterior primary teeth due to caries is considered a major issue due to the difficulty in regaining the esthetic crowns and attaching them until the normal exfoliation time. There have been many attempts and tools used to attach the composite crowns to the treated canals of primary anterior teeth. The study evaluates endodontic treatment for destructed primary maxillary incisors with a glass fiber-reinforced post as a retentive tool to hold the esthetic composite crowns until the normal exfoliation time of primary incisors. *Case Presentation*. A four-year-old child attending a dental clinic complained of pain of maxillary incisors. Dental examination showed irreversible pulpitis of four maxillary incisors indicated for root canal treatment and crown placement. Endodontic treatment was carried out, and a glass fiber-reinforced post was used to get successful retention for the composite crowns. Follow-up was carried out for 3 years. The 3 crowns were retained successfully until replaced by permanent incisors. One crown fell during the treatment course.

**Conclusion:**

Retention of primary teeth is one of the challenges in pediatric dentistry. Restoration of primary decayed incisors is important for child medical, physical, and psychological conditions. A glass fiber-reinforced post and core is a strong retentive tool for composite crown retention for primary incisors. This procedure opens the door for a strong tool to retain composite crowns for a long time. The glass fiber-reinforced post and core is a strong retentive tool for composite crown retention for primary incisors.

## 1. Background

The restoration of primary anterior teeth represented a great challenge in dentistry [1]. Many factors affect the treatment outcome of the restoration of primary teeth incisors and laterals: teeth, small size, child behavior cooperation, child's age, and cost of the treatment. Dental caries is the main causative factor in tooth structure destruction. The thin enamel and dentin layer enhance caries extension and tooth destruction with or without pulp involvement. Child behavior affects the selection of the treatment options and the treatment setting: either in a local environment or in treatment under general anesthesia [2]. The age factor will put the shadow of selecting the treatment with longevity rather than the option for the short term. With all the previously mentioned factors, the treatment cost will guide the parents to the treatment option, especially with the absence of dental insurance.

The maxillary primary incisors are the anterior primary teeth most affected by dental caries [1, 3, 4]. The caries pattern may affect all surfaces in a short time, leaving the tooth with the complete destruction of the tooth structure with or without pulp involvement. Esthetic satisfaction and the longevity of the restoration of anterior primary teeth were the main concerns for the parents and the dentist [5]. According to the American Academy of Pediatric Dentistry (AAPD), restoration techniques for the anterior primary teeth include the following: open-faced stainless steel crowns, full-coverage composite, strip crowns, and full white ceramic crowns [6].

The most conservative treatment (in primary and permanent dentition) is a direct restoration performed with resin-based composites. This treatment has always been preferred due to it being esthetically pleasing [7], reparable [8, 9], conservative [4], and economically affordable. Direct restorations can also be performed in an endotreated tooth with success following basic principles [4]. In addition to the revised challenges, restorations could be very difficult when the crown structure is destroyed completely with pulp involvement as well as for younger ages. For conditions that represent poor retention and poor oral hygiene, there will be no benefit to proceeding with this challenge. The best treatment will be extraction and replacement with kiddie partial [2, 7]. However, some parents' wish to keep the affected teeth may play a big role in going through this challenge. The main concern of anterior primary teeth restorations with root canal filling is the retention of the coronal restoration to the remaining tooth structure and the root canal filling [4]. This concern is the same for permanent dentition, and several classifications have been proposed for restoration according to the residual structure [10], and fiber posts appear to be the best build-up retention procedure [11].

The absence of primary anterior teeth for children in their young ages will affect their eating, smiling, pronunciation, and psychological interaction with others. Oral health neglect could be pronounced, but in many conditions, most of the parents prefer to keep the primary teeth instead of removing them and ask for alternative treatment rather than extraction of the anterior primary teeth. The present condition represents a special challenge where the dentist prepares four root canals with special retentive mean and coronal restoration for the maxillary incisors and laterals with a follow-up for 3 years until the eruption of the permanent incisor teeth [1, 4, 7].

## 2. Case Presentation

A 4-year-old female patient attended a pediatric dental clinic at the Dental College of Ajman University on September 2016. The patient was complaining of dental pain in the anterior maxillary teeth. The parents asked for a treatment to relieve the dental pain and restore the esthetic appearance for the patient. A dental examination showed destructed anterior maxillary teeth and bad oral hygiene of the remaining teeth. The dental history explained the bad oral hygiene and destructed anterior teeth due to bottle-feeding for 3 years and parental neglect in addition to the expensive dental treatment, which the parents could not afford. The dental examination showed retained roots of 51, 52, 61, and 62. The 62nd root showed great mobility and a bad prognosis for any future restoration. The intraoral radiograph revealed pulp involvement of 51, 52, 61, and 62 and showed root resorption.

## 3. Treatment Plan

The dentist decided to do a root canal filling for 51, 52, and 61 with fiber post and strip composite crown and extraction of 62. Labial and palatal infiltration was carried out for 51, 52, and 61, and gross carious lesions were removed. The pulp chamber was opened, pulp tissue was extirpated, and working length determination was carried out by IAPA film. The preparation of the canal was carried out for 51, 52, and 61, and proper irrigation with 2.5% of NaOCl and normal saline was performed for all the canals. The canals were dried using paper points, and the thick endo paste of Vitapex was used to fill the canals as much as possible and condensed by a Lentulo spiral into the canal; the opened canals were closed with a temporary filling. After 1 week, the patient came back for the dental appointment and the preparation of the fiber post space.

The fiber post preparation required the removal of 4-5 mm of the endo paste from the canal and cleaning with saline and dried with air. The spaces made for the post were acid etched, rinsed, and dried; a light-cured bonding agent was brushed on the etched surfaces and dispersed by an air blast. Before the placement of the fiber post, flowable composites were inserted in the post spaces, and then, the post was introduced to the space with the composite inside. Then, both were light-cured for 60 seconds.

**Figure 1:**
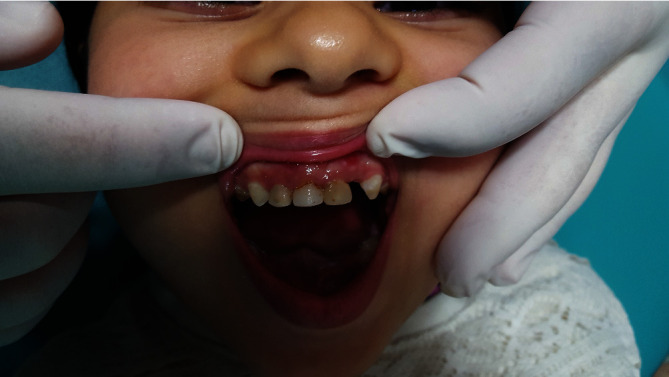
Final coronal restoration.

**Figure 2:**
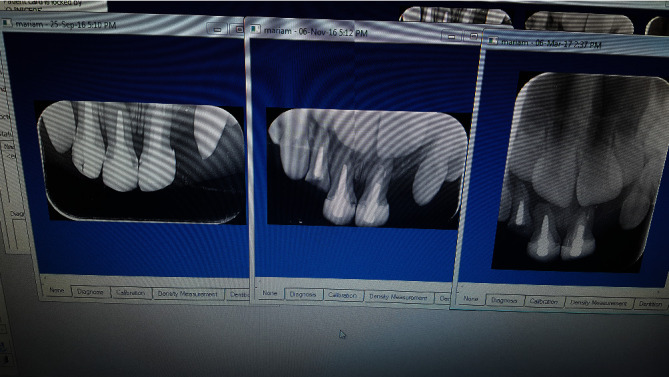
Radiographic follow-up of treated teeth.

The coronal area was etched, washed, and bonded using a light cure. Strip crowns were prepared according to the final post placement and filled with composite. Next, they were placed on 51, 52, and 61. Then, the final coronal restoration was cured with a light cure, 62 was extracted, and an IOAR was taken to keep the follow-up record (Figures 1 and 2).

**Figure 3:**
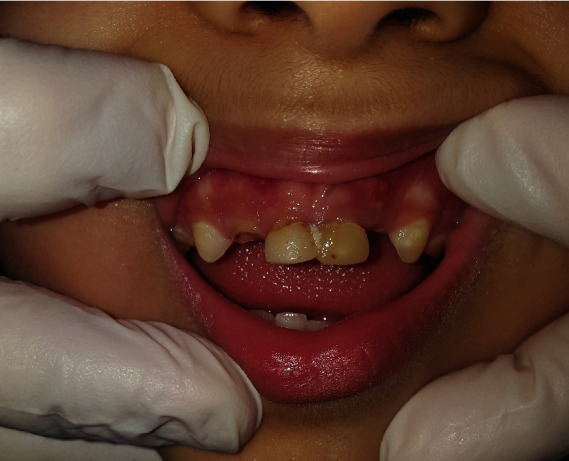
Clinical crowns of central incisors.

The patient returned after 2 months for IOAR and a checkup, and the parents were satisfied with the result with no pain or discomfort. However, the crown of 52 fell and was lost. The canal obturated, and the efforts for repeating the crown failed (Figure 3).

**Figure 4:**
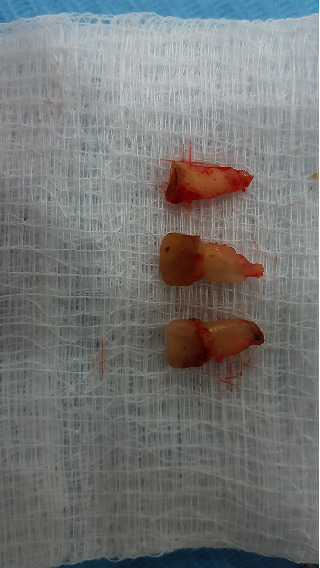
Extracted anterior primary teeth.

**Figure 5:**
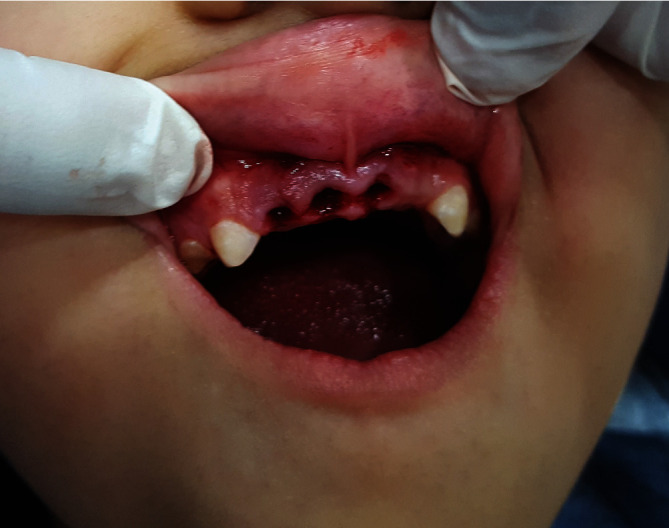
Extraction of primary anterior teeth.

On March 2017, the patient returned for another visit, and the dentist took IOAR. The radiograph showed evidence of normal physiological root resorption with no complaint or mobility. On June 2018, the patient complained of the mobility of the three teeth, which required the extraction of these teeth since it caused problems for her during eating (Figures 4 and 5).

**Figure 6:**
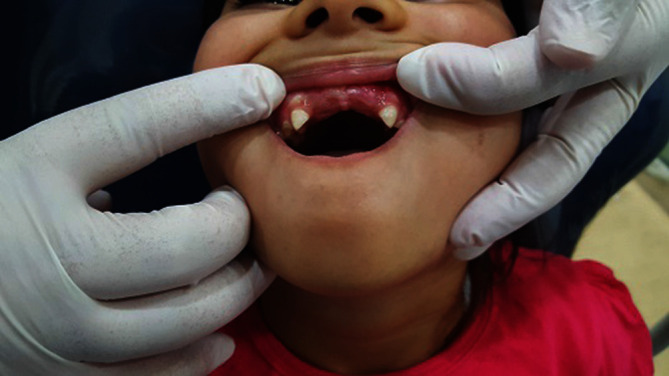
Clinical healing of anterior maxillary area.

On July 2018, the patient came with complete healing of the anterior maxillary area (Figure 6).

**Figure 7:**
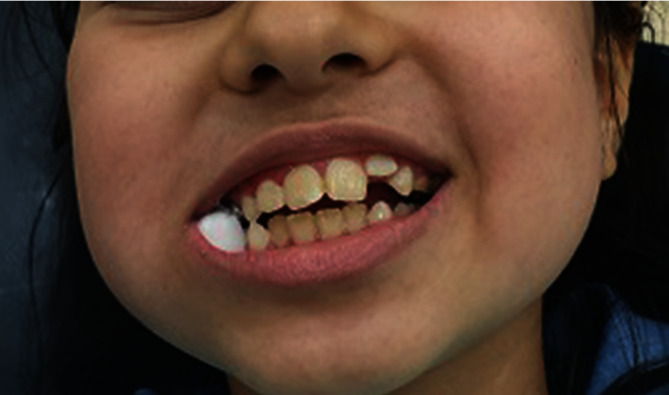
Eruption of anterior permanent incisors.

The next visit was on November 2018. Her centrals erupted. The patient's last visit was on May 2019 when the four anterior teeth, both maxillary centrals and laterals, fully erupted in the oral cavity (Figure 7).

## 4. Discussion

Esthetic restorations of primary anterior teeth represent a great challenge for most pediatric dentists. Small crown size, caries pattern, and the behavior of the patient plays a big role in successful treatment for restoring the esthetic appearance for young patients. Destructed anterior primary teeth with root canal treatment need a retentive post to hold the coronal crown on the treated canal [1, 2, 12, 13]. However, post selection is not an easy or successful procedure. Omega-shaped orthodontic wires and nickel-chromium cast posts did not meet the esthetic requirements as well as the adaptation to the canal shape [1]. A resin composite post building up directly did not achieve the required retention for the long term.

A glass fiber post is one of the posts used in some cases of primary anterior teeth [2, 13]. With root canal treatment and coronal placements, it can be used as an alternative to other post types for root canals. It has great advantages over the other posts, with greater flexural strength, easy application and handling, applications in high stress-bearing areas, esthetically acceptable, and the ability to bond to any composite [14]. According to the present study, it shows great retention within the canal for a long time. Due to these reasons, this method is considered superior to other types of posts for primary anterior teeth [15–18]. By the end of the treatment, parents were so happy with the results and on how we could keep the teeth until the permanent teeth erupted without affecting the child's appearance.

Restoration of child esthetics became one of the main concerns for parents as well as for children. Improving root canal post selections for a root canal-treated tooth will make the restoration of the coronal crown a successful one. The treatment of this case and the follow-up at 3 years until the primary is removed on time. The case has interesting, promising results for future treatments for traumatic or carious anterior primary teeth.

## Data Availability

No data were used to support this study.
